# Notes and News

**Published:** 1888-06-23

**Authors:** 


### NOTES AND NEWS.

Collected and Communicated.

On the 8th inst. the Italian Chamber of Deputies approved almost unanimously of the abolition of capital punishment.

Two hundred and eighteen candidates presented themselves at the Apothecaries' Hall for the examination in Arts on the 1st and 2nd inst.

It is proposed to erect a Women's Hospital at Calcutta, to take the place of the Countess of Dufferin's Fund dispensary in Circular Road. The cost is estimated at 40,000 rupees.

The collection at St. Barnabas Church, Addison Road, W., amounted, on Hospital Sunday, to £150 12*s.* 4*d.* Last year the sum received was £139 9*s.* 7*d.*

On account of the lamented death of the Emperor of Germany, the opening of the new building of the Great Northern Central Hospital by the Prince and Princess of Wales has been postponed for a few days. Due notice of the deferred date of the ceremony will be given.

Mr. C. W. Thies has been appointed secretary to the Royal Free Hospital, out of a large number of applicants. Mr. Thies has had a sound business training, and his devoted labours as honorary secretary to more than one philanthropic and charitable work has given him an experience which cannot fail to prove valuable to him in his new position.

H.R.H. the Princess Louise (Marchioness of Lorne) has graciously consented to be present at a flower show and musical conversazione, to take place at the National Hospital for the Paralysed and Epileptic (Albany Memorial), Queen-square, Bloomsbury, on Thursday, the 5th July. Mr. and Mrs. Herschel, Mrs. Lemon, Madame Norman-Neruda, and Mdlle. Janotha will assist. There will be a display of roses by Messrs. Wm. Paul and Sons, and of cut flowers by Mr. T. Ware.

Mr. Albert L. Boyce, the secretary of the Norfolk and Norwich Hospital, has been elected secretary superintendent to the Royal United Hospital, Bath. There were forty applicants, and Mr. H. J. Collins, of the Hospitals Association, and Captain Hincks, of the Gordon Hospital for Fistula, were the other selected candidates. As the Norfolk and Norwich Hospital holds a deservingly high place amongst provincial hospitals, we can cordially congratulate the United Bath Hospital Committee and Mr. Boyce. The traditions of the United Bath Hospital are unusually good, one of the most able and successful of hospital superintendents—Mr. W. L. Saunder, of the Manchester Royal Infirmary, having commenced his career and training at Bath.

The articles on hospital topics which appeared in the *Times* during the Hospitals Week, have been reprinted in pamphlet form, and can be obtained by applying to the Secretary of The Hospitals Association, Mr. L. Broke Willoughby, Norfolk House, Norfolk Street, Strand.

On behalf of the funds for the establishment of a hospital in Leicester, for children, in aid of which a fête is to be held in the Abbey Park on the 28th inst., the Mayor (Councillor T. Wright) has prepared a statement showing the pressing need of such an establishment.

Lord Colchester and Mr. H. H. Grenville Wells have become life members of The Hospitals Association, and the following gentlemen have become annual members: Mr. A. Wynn Corrie, Captain Griffiths, Mr. Edgar Hanbury, Mr. Edmund S. Hanbury, Dr. A. Symons Eccles, Mr. Adrian Hope, and Captain Gurney Hanbury.

Canon Duckworth, lecturing at St. Philip's, Waterloo-place, on the afternoon of Hospital Sunday, proposed the institution of a tax to provide medical relief for the poor of London. Perhaps the increasing necessities of our hospitals may bring this about; but it will be an indelible disgrace to the people of London if it is left to the State to force them to be charitable by Act of Parliament.

Miss Rawson has promised to bear the greater part of the cost of the new convalescent home at Harrogate, now in course of construction. The cost of the home when completed will be about £10,000, of which £1,000 has been given by an anonymous donor; the remaining expense will be borne by Miss Rawson, whose name the home is to bear. The Earl of Harewood has given a pine wood plantation at the back of the site for the use of the patients as a recreation-ground.

The work of The Hospitals Association is being carried on with great energy and success, and many influential persons interested in the administration of hospital affairs and finance are joining the association. It is hoped that all who are in any way connected with hospitals, or who are interested in, or responsible for, the management of hospitals and nursing institutions will join this important and useful association. Special facilities have recently been made whereby ladies on the staff of hospitals and nursing institutions can become members, the annual subscription for these being 10*s.* 6*d.*, instead of the ordinary annual subscription of one guinea.

A special meeting of the governors of Cork District Lunatic Asylum was held recently for the purpose of taking into consideration the inadequate accommodation in the asylum at present. From the report of Dr. Dwyer, Resident Medical Superintendent, it appears that there are 108 inmates in the asylum over the certificated accommodation, which included the hospital. Should the latter, which contains 100 patients, be excluded, there would be close on 200 patients over the certificated accommodation. Dr. Dwyer suggests that, when the additional building is constructed, it might be worth trying, in a small way, to copy the Royal Scotch Asylums, by converting a portion of it into accommodation for a better class of patients, whose friends would be prepared to pay according to the comforts bestowed on them. This would be a boon to people who object to private asylums, and the rates would not suffer, as the paying wards would more than defray their annual expenses. After discussing the matter, and inspecting the sites proposed, the governors directed plans to be prepared for the erection of the building, to contain 300 persons, with one side for males and one for femal.s.

THE following vacancies are announced this week, the amount of salary in each case being given after the name of the institution :—

**Resident Medical Officers.**

Croydon Union. Assistant Medical Superintendent and Dispenser at the Infirmary, May Day Road, Croydon.—£125, increasing by £5 per annum to £150, with board, etc.
Edinburgh City Poorhouse, Craig lockhart. Resident Medical Officer.—£80 per annum, with board.
Evesham Joint Hospital Board. Superintendent General Manager for the Evesham Sanatorium.—£30 per annum, with board.
Guest Hospital, Dudley. Resident Medical Officer.—£110 per annum, with board, etc.
Hospital for Consumption and Diseases of the Chest, Brompton. House Physician.
North Eastern Hospital for Children, Hackney Road, E. Junior House Surgeon.—£30 for six months.
Prison Commissioners, Scotland. Resident Surgeon for one of H. M. Prisons in Scotland.—£200 per annum, with increase of £50 after five years, and further increase of £50 after ten years.
Ramsgate and St. Lawrence Royal Dispensary and Seamen's Infirmary. Resident Medical Officer.—£120 per annum.
West Sussex, East Hants, and Chichester General Infirmary and Dispensary. House Surgeon and Assistant Secretary.—£100 per annum, with board.
Wolverhampton and Staffordshire General Hospital, Wolverhampton. Resident Assistant.—Board, no salary.

**Non-resident Medical Officers.**

Evelina Hospital for Sick Children, Southwark Bridge Road, S.E. Surgeon to Out patients.
Parochial Board of Pennygorm and Torosay. Medical Officer.—£100 per annum.

A SERVICE will be held in Westminster Abbey at three o'clock on Thursday, the 28th inst., in commemoration of the Jubilee of her Majesty's Coronation in the Abbey, and in order to associate a charitable object with the service, a collection will be made in aid of the funds of the Westminster Hospital. The north transept will be open to the public. Admission to the rest of the Abbey will be by tickets, which can be obtained from the secretary of the Westminster Hospital.

THE *Aberdeen Evening Gazette* takes occasion to remind the working men there of the privileges conferred upon them by the new act of incorporation of the Royal Infirmary. Artizans following any trade, whether as employés of the same firm or not, may nominate an "annual manager" for every £15 they collectively contribute, and a "life manager" for every £50. The *Gazette* has done well to point this out to the Aberdeen workmen, as many of them might never have heard of it otherwise, and the knowledge will doubtless greatly increase their interest in the infirmary and its management.

IN these days, when so much is said about the deterioration of the servant class, especial interest attaches to such a meeting as was held at the Ladies' Charity School, Powis Gardens, Notting Hill, on Saturday, June 9th. As many of the old scholars as can manage to come meet there annually on that day (being the anniversary of the opening of the school in 1702), and, judging by the cheerful conversation that goes on, this meeting of old schoolfellows is a very great treat. The ladies of the committee attend to give the girls tea, and to hear how each one is getting on, and rewards are given for length of service. On this occasion, amongst those who received prizes for five years' service were two young women who had remained in the same family to whom they first went on leaving school at the age of fifteen. This is a good record. There is a Samaritan Fund attached to the school, to which the old scholars are encouraged to subscribe, and through it, if they do not want help themselves, they are enabled to help their old friends who are less fortunate. The Ladies' Charity School is supported by voluntary contributions, and receives the daughters of respectable parents in reduced circumstances by election, or on payment of twenty guineas annually, and trains them as domestic servants.

WITH a view to carrying out the resolution adopted by the House of Commons in March, 1886, that immediate steps should be taken to ensure the full and accurate collection and publication of labour statistics, the Secretary of the Board of Trade has just sent out a special form to the secretaries of hospitals, asking for full information as to the remuneration of all hospital employés.

COVENTRY, which is already rich in charities, benefits largely by the will of Mr. David Spencer, wool merchant, of that city, who died a few days since. After giving £30,000 to Miss Scampton, a niece, and large sums to other relatives, he bequeaths £20,000 for technical instruction, £2,000 to the Coventry and Warwickshire Hospital, £500 each to a dozen religious and philanthropic societies, and the residue for the benefit of aged men and women, citizens of Coventry.

SMALL-POX has broken out a Preston, and is spreading with great rapidity, 185 houses having been officially registered at the sanitary department of the corporation as containing patients suffering from small-pox. It is estimated that there are now 200 cases in the town, all of which have developed in less than a week. This epidemic, and the rapidity with which it is spreading should be a warning to the good people of Nottingham to talk less and do more in the matter of erecting a hospital for infectious diseases in their town.

THE late Mr. George Sturge, of Woodthorpe, Sydenham Hill, who died in April last at the great age of ninety years, executed a deed of trust in November, 1883, by which he vested in trustees upwards of £300,000, and directed them, after providing for life annuities to numerous relatives and friends, to pay in instalments the sum of £95,500 to various charitable, philanthropic, and religious institutions, eighty-three in number, in certain specified shares, and on or before January 1st, 1901, to divide the residue of the trust funds between six specified charitable and religious institutions.

THE annual sale of invalids' work was held at the Northern Counties Home for Incurables at Mauldeth, near Manchester, on the 13th inst. The work exhibited at the stalls is all done by the patients, and the proceeds of the sale do not go towards the funds of the institution, but in part towards maintaining the supply of fabrics and other materials of which the goods are made, and in part to the private resources at the disposal of the individual patient. The sale is thus a thing apart from the general work of the hospital, which is carried on solely by voluntary contributions and donations, the inmates being elected by the vote of the subscribers. The building is beautifully situated among extensive grounds laid out with lawn and shrubbery, and the home has an air of substantial comfort about it.

ARRANGEMENTS are being made for the present year's Hospital Saturday collection on a larger scale than in former years. Already between twenty thousand and thirty thousand collection sheets have been addressed to the principal industrial establishments and warehouses of London, special efforts being made to increase what is called the workshop collection during the present year. The council of the fund are not satisfied with the success achieved in this department of their work, as hitherto the workshop collection has not compared favourably with provincial collections. Taking into consideration the difference in the population, the amount contributed by the working people in Birmingham is considerably more than the contributions from the same class in London. The street collection in London which has steadily increased from £258 in 1874, to nearly £5,000 last year, will, it is expected, show a still further increase during the present year, as a larger number of promises of assistance from ladies and tradesmen are to hand than at any former collection. It is expected that every railway station and every leading thoroughfare in the metropolis will have its collector on July 14th next., this being the date fixed for the out-door collection. Lord Brassey, president of the fund, writes : "He does not think that a collection of £50,000 is too much toward the support of the hospitals and dispensaries of London."

